# Role Overload and Work Performance: The Role of Psychological Strain and Leader–Member Exchange

**DOI:** 10.3389/fpsyg.2021.691207

**Published:** 2021-05-21

**Authors:** Wei-Gang Tang, Christian Vandenberghe

**Affiliations:** Department of Management, HEC Montréal, Montréal, QC, Canada

**Keywords:** role overload, work performance, leader–member exchange, psychological strain, depression

## Abstract

The relation between role overload and work performance remains insufficiently understood. Drawing upon conservation of resources theory, we expected role overload to negatively relate to performance through psychological strain and this relation to be buffered by leader–member exchange (LMX). Study 1 (*N* = 212) examined depression as a severe type of strain that mediates between role overload and in-role performance, job dedication, and voice behavior. Study 2 (*N* = 191) used generic, perceived strain as a mediator between role overload and in-role performance and reward recommendations. Both studies tested LMX’s buffering effect, controlling for role ambiguity and conflict. A supplementary panel study (*N* = 99) assessed the temporal relationship between role overload and strain. Role overload triggered psychological strain, which undermined performance, and LMX acted as a buffer on role overload, but not on role ambiguity or role conflict. We discuss the implications of these findings for theory and practice.

## Introduction

Roleoverload,^1^ a work condition where people perceive role demands as exceeding their time, energy, and capabilities (Rizzo et al., 1970), looms ever larger in the workplace nowadays, inflicting significant costs on employees and organizations (Alfes et al., 2018). Role overload is associated with an array of negative consequences such as psychological strain (Glazer and Beehr, 2005), turnover intention (Jensen et al., 2013), reduced organizational citizenship behaviors (OCBs; Eatough et al., 2011), lack of organizational commitment (Fisher, 2014), and low work performance (Gilboa et al., 2008). In parallel, the workplace becomes increasingly driven by performance due to the escalating global competition (Tsui, 2007). Work performance has been established as the key yardstick by which employees are evaluated and rewarded, for it is the cornerstone of the organization’s success. Thus, it is important for management scholars and practitioners to understand how and when role overload impacts work performance.

Although role overload and work performance have been long studied, the mechanisms underlying this relationship and associated boundary conditions remain insufficiently understood. Regarding the mechanisms, we argue that psychological strain is a potential pathway through which role overload undermines work performance. Drawing on conservation of resources (COR) theory (Hobfoll, 1989, 2001), we consider role overload taxing because it reflects the perception that situational demands exceed one’s personal resources. As such, role overload can trigger a variety of stress reactions, ranging from mild forms of psychological strain such as anxiety (Mazzola and Disselhorst, 2019) to more severe ones such as depression (Beehr et al., 2000). In line with COR theory, psychological strain captures the resource depletion process in which employees feel a significant loss of energy and resources (Halbesleben et al., 2014). Thus, role overload may act as a hindrance stressor that triggers psychological strain, which would ultimately impede work performance (LePine et al., 2005).

Prior studies regarding potential boundary conditions of role overload are scarce. Although stress theorists have long emphasized the need to “specify the conditions under which some stimuli are stressors” (Lazarus and Folkman, 1984, p. 21), few researchers have examined the boundary conditions that specifically apply to role overload. As some studies have reported the relation between role overload and performance to be negative while others have reported it to be positive (e.g., LePine et al., 2004; Gilboa et al., 2008; Bowling et al., 2015), boundary conditions likely operate. We contend that leader–member exchange (LMX; Dulebohn et al., 2012), which refers to the quality of the exchange relationship between the employee and the leader, moderates the relationship between role overload and psychological strain, ultimately affecting work performance. From a COR theory perspective (Hobfoll, 2001), high-quality LMX constitutes a social context abounding in opportunities and resources, which feeds the resource pool of employees (e.g., Ozer et al., 2014), suggesting that LMX can enable employees to better deal with their workload (Harris and Kacmar, 2005). We thus expect LMX to weaken the positive relationship between role overload and psychological strain, thereby protecting work performance.

**FIGURE 1:**
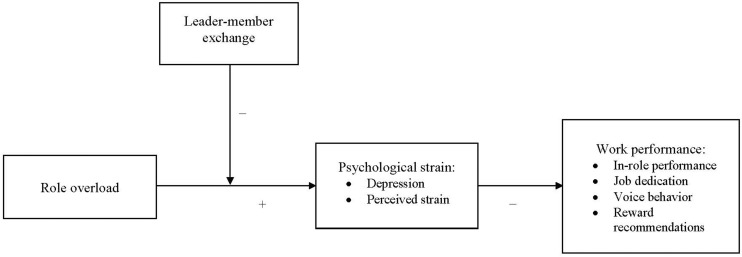
Theoretical Model for the Study.

Our research makes four major contributions. First, we examine a wider spectrum of strain mechanisms than did prior research by which role overload undermines work performance. This endeavor answers Gilboa et al.’s (2008, p. 256) call for more studies to explore the “mechanisms (mediators) through which role stressors affect performance.” In doing so we expand the role stress literature by adding depression as an essential strain pathway, thus substantiating the notion that if the imbalance between role demands and resources tilts too much toward the demands, people may fall victim to depression, which in turn will hurt their functioning (e.g., job performance). Second, to enrich the understanding of the scope of consequences role overload can induce, we examine multiple aspects of work performance (in-role performance, job dedication, voice behavior, and reward recommendations). Prior studies mainly zeroed in on single employee outcomes like innovative behavior (Montani et al., 2020) and mental health (Alfes et al., 2018). Our research extends previous work by demonstrating that role overload can undermine multiple aspects of performance-related outcomes. Third, we demonstrate that LMX represents an important relational context that can buffer the hindering effects of role overload. According to COR theory, LMX constitutes a reservoir of resources available to employees (Beehr et al., 1990). Fourth, we show that the moderating effect of LMX applies only to role overload, but not to role ambiguity and role conflict, thereby illustrating the sensitivity of role overload to resource-providing contexts. In the following sections, we present our hypotheses and research model (Figure 1).

## Theory and Hypothesis Development

### Role Overload, Psychological Strain, and Work Performance

Among the various work stressors that have been studied, the trilogy of role stressors—namely role overload, role ambiguity, and role conflict—has emerged as a prominent typology from the perspective of role theory (Kahn et al., 1964; Rizzo et al., 1970), which posits that employees are organized to fulfill requisite roles (i.e., interdependent, recurring behaviors; Katz and Kahn, 1978) for the going concern of the organization. It is the recurring interactions among individuals within and across different functions that give rise to stressful encounters, hence role stressors. Specifically, role ambiguity and role conflict refer to situations where the behaviors expected of an employee are unclear and contradictory, respectively (Rizzo et al., 1970), while role overload refers to situations where role demands exceed an employee’s resources such as time, energy, and capability (Eatough et al., 2011).

Unlike role ambiguity and role conflict which have been found to negatively associate with performance, several meta-analyses have reported role overload to be non-significantly related to performance (Örtqvist and Wincent, 2006; Gilboa et al., 2008; Eatough et al., 2011). The non-significant relationship can be explained by two factors: (a) role overload may be perceived as a hindrance or a challenge, which obscures the nature of its contribution to performance, and (b) competing mechanisms may be at play such as role overload positively affecting performance through enhanced motivation while negatively affecting it through increased strain. Although not examined in the context of role overload, these explanations are derived from the challenge-hindrance model of work stress (e.g., Crawford et al., 2010; Mazzola and Disselhorst, 2019).

We focus on the hindrance mechanism that presumably connects role overload to reduced work performance. Specifically, we posit that psychological strain represents a critical pathway through which role overload may affect performance. Psychological strain reflects adverse employee reactions that feature a strong sense of loss and lack of energy (Vuori and Vinokur, 2005; Vuori et al., 2012) and as such are affective in nature, such as job dissatisfaction (Spector et al., 2000) and negative emotions (Spector et al., 2000; Boswell et al., 2004). Embodying a resource depletion process in response to work stressors (Hobfoll, 1989; Grandey et al., 2012), psychological strain has drawn significant attention to its major aspects, such as emotional exhaustion (Ford et al., 2014; McCarthy et al., 2016) and depression (Dormann and Zapf, 1999; Tucker et al., 2005; Diestel and Schmidt, 2011). Among various aspects of psychological strain, depression exerts perhaps the most devastating effects on work performance (Diestel and Schmidt, 2011) and was thus selected, together with perceived strain, as the core components of psychological strain for the current research.

Role overload is a specific stressor that reflects the perception that the demands of one’s work role exceeds personal resources (Eatough et al., 2011). As such, role overload has the potential to give rise to resource depletion, a phenomenon that can be understood through the COR lens. COR theory posits that individuals seek to retain, protect, and create resources, and that stress reactions result from actual or anticipated resource losses (Hobfoll, 1989, 2001). The experience or the expectation of resource losses leads to a sense of depletion and lack of energy (Crawford et al., 2010; Halbesleben et al., 2014). Thus, the more severe the imbalance between role demands and resources inherent to role overload, the more critical the experience of resource loss and strain (e.g., ranging from general perceived strain to depression). Empirical studies have shown that role overload is associated with various aspects of psychological strain, such as increased job stress (Bolino and Turnley, 2005; Shultz et al., 2010), psychological distress (Jex et al., 2001; Rafferty and Jimmieson, 2010), job tension (Perrewé et al., 2005), anxiety (e.g., Glazer and Beehr, 2005), and depression (Beehr et al., 2000). Moreover, when individuals devote time and resources to dealing with overwhelming role demands, concurrently they lack the resources required to complete in-role duties, let alone the extra-role behaviors that benefit the organization (LePine et al., 2005). Indeed, role overload has been found to be related to increased psychological strain (de Croon et al., 2004; Örtqvist and Wincent, 2006) while the latter has been shown to be negatively related to work performance (Ford et al., 2011). Drawing upon COR theory (Hobfoll, 1989, 2001), we thus contend that psychological strain constitutes a key resource-depletion mechanism through which role overload may undermine work performance. Moreover, as role overload is distinct from the other two role stressors (Eatough et al., 2011), we expect our contention to hold while controlling for role ambiguity and role conflict. Thus, we give the following hypotheses.

*Hypothesis 1a*: Controlling for role ambiguity and role conflict, role overload is positively related to psychological strain.
*Hypothesis 1b*: Controlling for role ambiguity and role conflict, role overload is indirectly, negatively related to work performance through increased psychological strain.

### The Moderating Role of LMX

The role stress literature has seldom considered the role of context in shaping the effects of role overload. We contend that contextual characteristics that can create opportunities would make role overload less hindering. In one of the few attempts that looked at the influence of social context on role overload’s effects, Fisher (2014) found empowerment practices and cooperative climate to buffer the negative relationship between role overload and affective commitment. These effects were explained by the increased resilience provided by empowerment practices and the availability of psychological and instrumental resources afforded by cooperative relations with others. Of incidental interest as well is another study (Dormann and Zapf, 1999) that addressed the longitudinal effects of social stressors (assessed through a general measure of irritating work events) and found supervisor support to mitigate the positive relationship of social stressors to depressive symptoms.

According to COR theory (Hobfoll, 1989, 2001), a relational context that feeds individuals’ resources has the potential to mitigate the effect of role overload on psychological strain. Such context can be described through the quality of LMX relationships. Guided by role theory, LMX research (Graen and Uhl-Bien, 1995; Dulebohn et al., 2012) has shown that individuals in high-LMX relationships enjoy valued advantages such as trustful relationships with, and emotional support from, the leader, as well as more rewards. These individuals may experience role overload as less hindering thanks to the resources available to them in handling role demands (Harris and Kacmar, 2005). For instance, they may expect being rewarded when meeting the expectations of their jobs. In contrast, individuals in low-LMX relationships do not receive the same advantages, are confined to narrowly defined roles, and receive assignments with little decision latitude (Liden et al., 1993). Therefore, they are more likely to experience role overload as hindering because they anticipate resource losses while dealing with role demands with little hope of receiving support that would sustain their effort. Moreover, these individuals have few rewards to expect even when they handle role demands successfully.

We were not able to locate any study that addressed the moderating role of LMX between role overload and psychological strain. However, at least two studies warrant a mention because even though they did not assess role overload *per se*, they looked at LMX as a moderator between social stressors and strain. Harris and Kacmar (2005) found that perceived politics (i.e., a social stressor) was less strongly associated with psychological strain (measured through anxiety) among employees reporting high LMX. In contrast, another study (Hesselgreaves and Scholarios, 2014) found no evidence for LMX to buffer the straining effect of role demands. However, that study used an undifferentiated measure of role demands that contained a variety of stressful conditions. Finally, one study (Harris et al., 2008) reported that LMX mitigated the relationship between strain and turnover intention. It must be noted that, according to COR theory (Hobfoll, 1989, 2001), it is the relationship between role overload and strain— but not between strain and outcomes—that LMX should moderate. This is because LMX serves to build individuals’ resources in the face of role overload.

By the above reasoning, we posit that when enjoying high LMX, employees tend to experience role overload less negatively, and thus will feel less psychological strain. Moreover, meta-analysis has shown that psychological strain variables (e.g., depression and general anxiety) have moderate-to-strong negative correlations with a variety of work performance criteria (Ford et al., 2011). Therefore, the moderating effect of LMX should extend to the indirect relationship between role overload and work performance through psychological strain. We also maintain that these effects will hold while controlling for the interactive effects of role ambiguity and role conflict with LMX. As our reasoning suggests LMX acts as a relational context offering resources to employees, it is important to show that LMX uniquely interacts with role overload. Specifically, role overload features a perceived imbalance between role demands and personal resources (e.g., Eatough et al., 2011), making it sensitive to LMX’s buffering effect; whereas role ambiguity and role conflict reflect stressors that do not speak to resource imbalance. The above reasoning leads to the following hypotheses.

*Hypothesis 2a*: Controlling for role ambiguity and role conflict, LMX moderates the positive relationship between role overload and psychological strain such that this relationship is less (vs. more) positive when LMX is high (vs. low).
*Hypothesis 2b*: Controlling for role ambiguity and role conflict, LMX moderates the indirect, negative relationship between role overload and work performance through increased psychological strain such that this relationship is less (vs. more) negative when LMX is high (vs. low).

## Overview of the Studies

To test our hypotheses, we conducted two primary studies, sampling customer-service employees in Canada, for this population of employees are reputed to be highly exposed to job stress and their performance tends to suffer from having to meet various expectations of multiple stakeholders (Netemeyer et al., 2005). Meanwhile, this population of employees are also sensitive to the exchange relationship with their supervisors (Eisenberger et al., 2014). Thus, participants in both studies made ideal samples for testing our hypotheses. In both studies, we used role overload and LMX as interactive predictors of psychological strain, operationalized through depression in Study 1 and perceived strain in Study 2. We also included role ambiguity and role conflict in both studies, controlling for their main effects and their interaction effects with LMX on psychological strain. Moreover, we examined various aspects of work performance rated by supervisors, namely in-role performance, job dedication, and voice behavior in Study 1, as well as in-role performance and reward recommendations in Study 2. As both studies were conducted in French, a translation-back-translation procedure was used to translate the English survey items into French (Schaffer and Riordan, 2003). Unless otherwise stated, items in both studies were rated using a 5-point Likert-type scale (1 = *strongly disagree*; 5 = *strongly agree*).

## Study 1 Method

### Sample and Procedure

We obtained agreement from the customer service departments of Canadian companies operating in various industries (telecommunications, electronic equipment, insurance, electricity, and marketing services) to participate in a study about leadership and performance. The number of employees per department ranged from 40 to 70 (*M* = 51.83; *SD* = 12.37). The employee questionnaires contained, among others, measures of role stressors, LMX, depressive symptoms, and demographics. Managers separately assessed employees’ in-role performance, job dedication, and voice behavior. A cover letter informed employees and managers of the study purposes, ensuring that responses would be confidentially treated. Questionnaires were coded so that employee and manager responses could be matched and were completed during work hours and later collected by the researchers. As a compensation for their time, employees received a $5 gift card while managers received a $30 gift card for rating employee performance. We collected 220 (out of 311) usable employee responses, for a 70.74% response rate (ranging from 48.57 to 84.44% across departments). The managers rated the performance of all employees, among whom 45.40% were female, average age was 34.73 years (*SD* = 7.97), average organizational tenure was 3.84 years (*SD* = 5.14), and average tenure with the manager was 2.46 years (*SD* = 2.98).

### Measures

#### Role Overload

We measured role overload (α = 0.75) using Schaubroeck et al. (1989) 3-item scale (e.g., “I never seem to have enough time to get everything done at work”).

#### Leader–Member Exchange (LMX)

We measured LMX (α = 0.92) using Liden and Maslyn (1998) 12-item scale, which comprises four dimensions: affect (3 items; e.g., “I like my supervisor very much as a person”), loyalty (3 items; e.g., “My supervisor would come to my defense if I were ‘attacked’ by others”), contribution (3 items; e.g., “I do not mind working my hardest for my supervisor”), and professional respect (3 items; e.g., “I admire my supervisor’s professional skills”).

#### Depression

We measured depression (α = 0.92) using Salokangas et al. (1994) scale of depressive symptoms. Respondents indicated the extent to which they experienced 10 depressive symptoms (over the past month), such as “feeling worthless,” “feeling blue,” “feeling a lack of energy,” or “not enjoying life” (Vuori and Vinokur, 2005). One item—referring to “sleeping disorders”— represented a somatic complaint, hence was dropped.

#### Work Performance

Managers rated employee performance along three dimensions. First, *in-role performance* (α = 0.92) was assessed by Williams and Anderson (1991) 7-item scale. This scale measures the prescribed aspects of job activities (e.g., “Adequately completes assigned duties”). Second, *job dedication* (α = 0.98) was measured by Van Scotter et al., 2000, p. 529) 6-item scale, which captures “effort, initiative, persistence, and self-discipline” (e.g., “Persists in overcoming obstacles to complete a task”). Third, *voice behavior* (α = 0.97) was measured by Van Dyne and LePine (1998) 6-item scale, which assesses the extent to which employees challenge the *status quo* by making suggestions for change (e.g., “develops and makes recommendations concerning issues that affect this work group”). Voice items were rated on a 5-point scale ranging from 1 (*never*) to 5 (*very often*).

#### Control Variables

We controlled for employee age, gender, organizational tenure, and tenure with the manager, for they were found to relate to depression (Eriksen and Kress, 2008) and performance (Wright and Bonett, 2002; Shore et al., 2003). Moreover, we controlled for role ambiguity and role conflict. We measured role ambiguity (α = 0.90) and role conflict (α = 0.90) using Rizzo et al. (1970) 5-item role clarity scale (reverse coded; e.g., “I know exactly what is expected of me”) and 8-item role conflict scale (e.g., “I work under incompatible policies and guidelines”), respectively.

## Study 1 Results

### Confirmatory Factor Analyses

We conducted confirmatory factor analyses (CFAs) to assess the distinctness of our variables, using Mplus 8.3 (Muthén and Muthén, (1998-2017)). Because our data were not multivariate normal, we based CFAs on the maximum likelihood estimation method (MLM), for it generates parameter estimates and fit indices that are more robust to multivariate non-normality (Byrne, 2012). Moreover, we used item-parceling method to maintain a favorable indicator-to-sample-size ratio (Little et al., 2013). Specifically, we parceled the nine items of depression into three indicators, using the balancing approach (i.e., adopting the high-to-low loadings procedure) that is suitable for unidimensional constructs (Little et al., 2013). As for LMX, we used facet-representative approach to parcel its twelve items into four indicators, representing affect, loyalty, contribution, and professional respect. We maintained the three original items as indicators for role overload. The hypothesized eight-factor model yielded a good fit to the data [χ^2^(247) = 353.16, CFI = 0.99, TLI = 0.97, RMSEA = 0.04, SRMR = 0.05] and outperformed all more parsimonious models (*p*s < 0.001) (Table 1), suggesting that our variables were distinct.

**TABLE 1:** Study 1 and Study 2 Confirmatory Factor Analysis Results: Fit Indices.

	χ^2^(df)	CFI	TLI	RMSEA	SRMR	Δχ^2^(Δ*df*)*^*a*^*
**Study 1 (*N* = 218)**						
(1) Hypothesized eight-factor model	353.16 (247)	0.99	0.97	0.04	0.05	–
(2) Combining role overload and depressive symptoms	496.22 (254)	0.95	0.94	0.07	0.07	139.35* (7)
(3) Combining depressive symptoms and LMX	698.02 (254)	0.90	0.88	0.09	0.11	332.53* (7)
(4) Combining role overload and LMX	Failed to converge					–
(5) Combining all three role stressors	902.37 (260)	0.86	0.83	0.11	0.11	535.04* (13)
(6) Combining in-role performance, job dedication, and voice behavior	1295.76 (260)	0.77	0.73	0.14	0.08	1031.76* (13)
(7) One-factor model	Failed to converge					–
**Study 2 (*N* = 199)**						
(1) Hypothesized seven-factor solution	259.55 (188)	0.97	0.96	0.04	0.05	–
(2) Combining role overload and perceived strain	379.11 (194)	0.92	0.91	0.07	0.07	127.78* (6)
(3) Combining perceived strain and LMX	455.29 (194)	0.89	0.87	0.08	0.10	216.67* (6)
(4) Combining role overload and LMX	Failed to converge					–
(5) Combining all three role stressors	660.41 (199)	0.80	0.77	0.11	0.12	405.12* (11)
(6) Combining in-role performance and reward recommendations	533.19 (194)	0.86	0.83	0.09	0.07	278.27* (6)
(7) One-factor model	1705.54 (209)	0.36	0.29	0.19	0.17	1465.98* (21)

CFI, comparative fit index; TLI, Tucker–Lewis fit index; RMSEA, root-mean-square error of approximation; SRMR, standardized root mean square residual.^*a*^When model comparison is based on MLM estimation, it is inappropriate to compute Δχ^2^ in the conventional way by direct subtraction (Byrne, 2012). To calculate the correct Δχ^2^, we applied Satorra and Bentler (2001) formula, which is also available on the Mplus website (http://www.statmodel.com/chidiff.shtml).* p < 0.001.

### Level of Analysis

Descriptive statistics and correlations are reported in Table 2. As individual data were nested within departments, it was necessary to ensure the data non-dependency. We computed intra-class correlation coefficients (ICC[1]) for core variables using one-way random effects analysis of variance (ANOVA) (Bliese, 2000). For role stressors, LMX, psychological strain (i.e., depression), job dedication, and voice behavior, the ANOVA result was non-significant. The result, however, was significant (*p* < 0.05) for in-role performance; yet the ICC(1) value was quite small (0.05)—an effect that is often considered negligible (LeBreton and Senter, 2008). Given weak group effects for core variables, we conducted the analyses at the individual level.

**TABLE 2:** Study 1 and Study 2 Descriptive Statistics and Correlations.

Variable	*M*	*SD*	1	2	3	4	5	6	7	8	9	10	11	12
**Study 1 (*N*s = 215–218)**														
(1) Age	34.73	7.97	–											
(2) Gender	1.55	0.50	0.32**	–										
(3) Organizational tenure	3.84	5.14	0.51**	−0.01	–									
(4) Tenure with manager	2.46	2.98	0.34**	−0.01	0.75**	–								
(5) Role ambiguity	2.17	0.95	0.08	−0.05	0.11	0.05	(0.90)							
(6) Role conflict	2.70	0.92	−0.01	−0.05	0.09	0.08	0.23**	(0.90)						
(7) Role overload	3.11	1.05	−0.13	−0.11	−0.14*	−0.06	0.06	0.36**	(0.75)					
(8) LMX	3.62	0.80	0.08	−0.02	0.12	0.14*	−0.44**	−0.16*	−0.11	(0.92)				
(9) Depression	2.31	1.07	−0.15*	0.07	−0.03	−0.09	0.24**	0.46**	0.33**	−0.27**	(0.92)			
(10) In-role performance	4.18	0.83	0.19**	−0.08	0.42**	0.38**	−0.15*	−0.01	−0.03	0.38**	−0.23**	(0.92)		
(11) Job dedication	2.92	1.25	0.13*	−0.12	0.28**	0.31**	−0.11	−0.04	0.04	0.27**	−0.19**	0.66**	(0.98)	
(12) Voice behavior	2.84	1.09	0.25**	−0.06	0.34**	0.30**	−0.20**	−0.03	−0.04	0.33**	−0.25**	0.55**	0.64**	(0.97)
**Study 2 (*N*s = 193-199)**														
(1) Age	33.28	7.01	–											
(2) Gender	1.52	0.50	0.31**	–										
(3) Organizational tenure	2.83	2.81	0.17*	0.05	–									
(4) Tenure with manager	1.99	1.78	0.04	−0.10	0.70**	–								
(5) Role ambiguity	2.05	0.87	−0.23**	−0.16*	−0.04	−0.02	(0.87)							
(6) Role conflict	2.53	0.87	−0.19**	0.00	0.09	0.07	0.19**	(0.84)						
(7) Role overload	3.08	1.02	−0.02	0.06	0.06	0.08	0.08	0.32**	(0.73)					
(8) LMX	3.65	0.73	0.04	0.09	−0.05	−0.04	−0.46**	−0.12	−0.05	(0.87)				
(9) Perceived strain	2.43	1.22	−0.10	−0.01	0.03	−0.01	0.16*	0.34**	0.28**	−0.23**	(0.90)			
(10) In-role performance	4.01	0.99	0.22**	0.15*	0.30**	0.23**	−0.25**	−0.17*	−0.05	0.19**	−0.35**	(0.93)		
(11) Reward recommendations	2.03	1.20	0.18*	0.13	0.14	0.22**	−0.23**	−0.22**	−0.04	0.15*	−0.29**	0.57**	(0.90)	

For Gender: 1 = female, 2 = male. LMX, leader–member exchange. Alpha coefficients are reported in parentheses along the diagonal.*p < 0.05, **p < 0.01.

### Hypothesis Testing

Table 3 reports the results of multiple regression analyses predicting psychological strain and performance. We mean-centered predictors before creating the interaction terms (Aguinis and Gottfredson, 2010). To predict psychological strain, we entered control variables in Step (i.e., Model) 1, role overload and LMX successively in Model 2 and Model 3, the interactions between LMX and role ambiguity and conflict in Model 4, and the LMX × role overload interaction in Model 5. To predict performance, we included controls, role overload, and psychological strain (i.e., depression) in Model 6s, then LMX and its interactions with the three role stressors in Model 7s.

**TABLE 3:** Study 1 and Study 2 Moderated Multiple Regression Analysis Results for Psychological Strain and Work Performance.

	Psychological strain					Work performance							
Variable	Depression (Study 1); perceived strain (Study 2)					In-role performance		Job dedication		Voice behavior		Reward recommendations	
	Model 1	Model 2	Model 3	Model 4	Model 5	Model 6	Model 7	Model 6	Model 7	Model 6	Model 7	Model 6	Model 7
**Study 1 (*N* = 212)**													
Age	−0.23**	−0.22**	−0.22**	−0.22**	−0.23**	–0.03	–0.03	0.06	0.05	0.11	0.10		
Gender	0.18**	0.20**	0.19**	0.19**	0.20***	–0.07	–0.06	–0.11	–0.10	–0.09	–0.06		
Organizational tenure	0.14	0.18	0.19*	0.18	0.20*	0.36**	0.34***	0.13	0.11	0.24*	0.24*		
Tenure with manager	–0.13	–0.15	–0.15	–0.15	–0.16	0.12	0.12	0.17	0.17	0.04	0.03		
Role ambiguity	0.15*	0.15*	0.09	0.09	0.08	–0.15	–0.04	–0.10	–0.01	−0.22***	−0.14*		
Role conflict	0.42***	0.35***	0.34***	0.34***	0.32***	0.04	0.04	–0.01	–0.01	0.04	0.05		
Role overload		0.19**	0.19**	0.19**	0.22***	0.06	0.07	0.13	0.14	0.09	0.11		
LMX			−0.13*	−0.14*	−0.15*		0.27***		0.19*		0.18*		
Role ambiguity × LMX				0.02	0.04		–0.04		0.04		–0.02		
Role conflict × LMX				0.00	0.06		0.07		0.01		0.03		
Role overload × LMX					−0.21***		–0.03		–0.04		–0.13		
Depression						−0.22**	−0.19*	−0.18*	−0.17*	−0.21**	−0.21**		
*R*^2^	0.27***	0.30***	0.32***	0.32***	0.36***	0.27***	0.34***	0.16***	0.19***	0.21***	0.25***		
Δ*R*^2^		0.03**	0.01*	0.00	0.04***								
Study 2 (*N* = 191)													
Age	–0.03	–0.03	–0.05	–0.05	–0.06	0.07	0.08					0.07	0.08
Gender	0.02	0.01	0.02	0.01	0.02	0.09	0.08					0.12	0.12
Organizational tenure	0.07	0.07	0.07	0.07	0.08	0.26**	0.27**					–0.07	–0.07
Tenure with manager	–0.09	–0.11	–0.11	–0.12	–0.13	0.05	0.05					0.30**	0.29**
Role ambiguity	0.14	0.14	0.05	0.03	0.05	−0.18**	–0.14					−0.17*	–0.15
Role conflict	0.34***	0.27***	0.26***	0.25***	0.22**	–0.06	–0.08					−0.15*	−0.18*
Role overload		0.22**	0.21**	0.22**	0.25***	0.02	0.05					0.03	0.05
LMX			−0.18*	−0.17*	−0.17*		0.05						0.00
Role ambiguity × LMX				–0.09	–0.07		–0.03						0.00
Role conflict × LMX				0.03	0.10		0.16*						0.11
Role overload × LMX					−0.18*		–0.05						–0.08
Perceived strain						−0.31***	−0.31***					−0.17*	−0.18*
*R*^2^	0.16***	0.20***	0.22***	0.23***	0.25***	0.29***	0.32***					0.21***	0.22***
Δ*R*^2^		0.04**	0.02*	0.01	0.02*								

Except for the R^2^ and ΔR^2^ rows, the values are standardized regression coefficients. LMX = Leader–member exchange. *p < 0.05, **p < 0.01, ***p < 0.001.

Hypothesis 1a predicted role overload to be positively related to psychological strain. As Model 2 shows, role overload was positively associated with psychological strain (b = 0.19, *p* < 0.01), controlling for demographics and the other two stressors. Hypothesis 1a is thus supported. Further, as Model 6s show, psychological strain was negatively linked to in-role performance (b = −0.22, *p* < 0.01), job dedication (b = −0.18, *p* < 0.05), and voice behavior (b = −0.21, *p* < 0.01). We used Hayes, 2018) PROCESS macro and the bootstrapping method to test Hypothesis 1b, which stated that role overload would be indirectly, negatively related to work performance through increased psychological strain. Specifically, we bootstrapped 5,000 samples to obtain 95% bias-corrected confidence intervals (CIs) (MacKinnon et al., 2004) for the indirect effects of role overload on performance through psychological strain. Results show that the indirect effects of role overload on in-role performance (−0.03, 95% CI = [−0.08, −0.01]), job dedication (−0.04, 95% CI = [−0.11, −0.01]), and voice behavior (−0.04, 95% CI = [−0.10, −0.01]) through psychological strain were significantly negative. Hypothesis 1b is thus supported.

**FIGURE 2:**
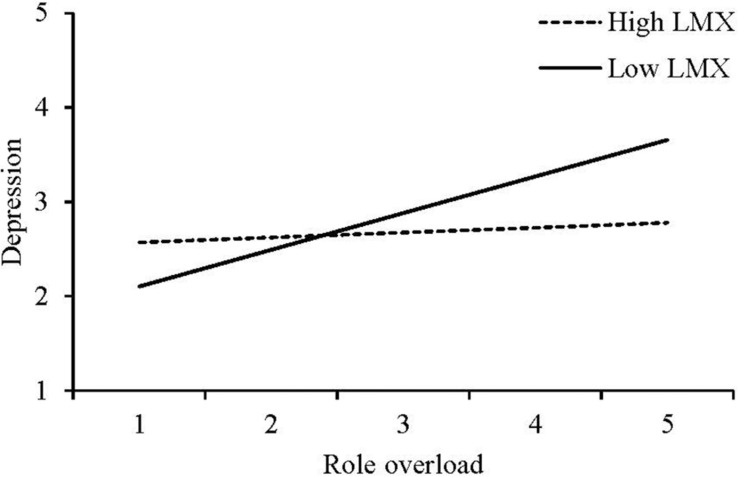
Study 1 Moderating Effect of LMX on the Relationship Between Role Overload and Depression.

Hypothesis 2a stated that, controlling for role ambiguity and role conflict, role overload would be less (vs. more) positively related to psychological strain at high (vs. low) LMX levels. As Model 5 shows, whereas LMX interacted with neither role ambiguity (b = 0.04, *ns*) nor role conflict (b = 0.06, *ns*), it did interact with role overload (b = −0.21, *p* < 0.001) in predicting psychological strain (i.e., depression). To illustrate this interaction, we plotted the regression line for depression on role overload at 1 *SD* below and above the mean of LMX (Aiken and West, 1991; Figure 2). Role overload was unrelated to depression at high LMX levels, *t*(211) = 0.59, *ns*, but was positively related to it at low LMX levels, *t*(211) = 4.38, *p* < 0.001, and the slope difference was significant: *t*(211) = −3.24, *p* < 0.01. Hypothesis 2a is thus supported.

Finally, Hypothesis 2b asserted that, controlling for role ambiguity and conflict, the indirect relationship between role overload and performance through psychological strain would be less (vs. more) negative at high (vs. low) LMX levels. We assessed these moderated mediation effects using PROCESS (Hayes, 2018), which was based on 5,000 bootstrap samples. Results show that the moderated mediation effect was significant for all performance outcomes: in-role performance (0.04, 95% CI = [0.01, 0.08]), job dedication (0.05, 95% CI = [0.01, 0.12]), and voice behavior (0.05, 95% CI = [0.01, 0.10]). Moreover, the indirect effect of role overload on in-role performance, job dedication, and voice behavior, was non-significant at high-LMX levels (−0.01, 95% CI = [−0.04, 0.02]; –0.01, 95% CI = [−0.06, 0.02]; and –0.01, 95% CI = [−0.06, 0.02], respectively) but significantly negative at low-LMX levels (–0.07, 95% CI = [–0.13, –0.02]; –0.09, 95% CI = [−0.20, −0.01]; and −0.09, 95% CI = [−0.18, −0.02], respectively). Hypothesis 2b is supported.

## Study 1 Discussion

Study 1 indicates that role overload negatively related to work performance through increased psychological strain, suggesting resource depletion is a central mechanism underlying this relationship. Results also show that LMX is an important relational context that mitigates the threatening potential of role overload, thereby preventing it from triggering the resource depletion process, which is detrimental to performance. This may occur because high LMX constitutes a supportive context ensuring that resources and rewards are available for employees to cope with role demands (Harris and Kacmar, 2005). As expected, role ambiguity and role conflict did not interact with LMX, suggesting that these role stressors may not be liable to the resource-providing influences of LMX. Study 2 aims to extend Study 1. To this end, it introduces another measure of psychological strain (i.e., perceived strain), as well as an alternative, performance-related outcome (i.e., reward recommendations), in addition to in-role performance.

## Study 2 Method

### Sample and Procedure

As in Study 1, we approached the customer service departments of Canadian companies that operated in various industries (telecommunications, electricity, and financial services) to participate in a study about leadership and performance. These departments employed between 34 and 60 employees (*M* = 42.33; *SD* = 9.52). A cover letter informed managers and employees of the research aim, ensuring that responses would be confidentially treated. During work hours, employees completed questionnaires about, among others, role stressors, LMX, perceived strain, and demographics. Managers separately rated subordinates’ performance and reward recommendations. Questionnaires were coded to allow matching employee and manager responses. From 254 prospective participants, we received 199 usable employee responses (for a 78.35% response rate; with response rates ranging from 60.00 to 87.50% across departments). Managers rated the performance of all employees, among whom 48.00% were women, average age was 33.28 years (*SD* = 7.01), average organizational tenure was 2.83 years (*SD* = 2.81), and average tenure with the manager was 1.99 years (*SD* = 1.78).

### Measures

We used the same scales as in Study 1 to measure *role overload* (α = 0.73; Schaubroeck et al., 1989), *LMX* (α = 0.87; Liden and Maslyn, 1998), and *in-role performance* (α = 0.93; Williams and Anderson, 1991); and *role ambiguity* (α = 0.87; Rizzo et al., 1970) and *role conflict* (α = 0.84; Rizzo et al., 1970) as control variables; with employee age, gender, organizational tenure, and tenure with manager as additional controls.

#### Perceived Strain

Following COR theory’s central tenet that strain is reflected in a feeling of having lost resources (or anticipating such losses) (Hobfoll, 2001), we developed a 3-item scale to measure such feeling. These items were “I have lost many resources (time, energy, and self-esteem) due to my work,” “I am undergoing a decrease of my general well-being due to my work,” and “My quality of life has been reduced by my work” (α = 0.90). We pilot tested this measure on a separate sample of employees from various organizations (*N* = 443), and examined correlations with depression, assessed via the same scale as in Study 1 (Vuori and Vinokur, 2005) (α = 0.95), and the General Health Questionnaire (GHQ-12; α = 0.86)—a well-established 12-item scale of psychological distress (Goldberg et al., 1997). Perceived strain correlated 0.63 with depression, indicating substantial convergence between the two variables. Moreover, perceived strain and depression strongly correlated with the GHQ-12 (*r* = 0.51, *p* < 0.001, and *r* = 0.66, *p* < 0.001, respectively), indicating that they both reflect an important distress component. This provides initial evidence for the validity of this newly developed measure of perceived strain.

#### Reward Recommendations

Managers assessed reward recommendations (α = 0.90) for their employees using Allen and Rush (1998) 5-item scale, which measures five organizational rewards (e.g., salary increase and promotion) on a 5-point scale (1 = *never*; 5 = *completely*).

## Study 2 Results

### Confirmatory Factor Analyses

As in Study 1, we first conducted CFAs with Mplus 8.3 (Muthén and Muthén, (1998-2017)) to assess the distinctness of study variables, by using the same item-parceling approach (Little et al., 2013). As Table 1 shows, the seven-factor model yielded a good fit to the data, χ^2^(188) = 259.55, CFI = 0.97, TLI = 0.96, RMSEA = 0.04, SRMR = 0.05. Moreover, it outperformed all alternative models (*p*s < 0.001). Thus, the constructs were distinct.

### Level of Analysis

Descriptive statistics and intercorrelations for the study variables are reported in Table 2. Like Study 1, Study 2 had nested data (individuals being nested within departments), raising a non-independency concern. We followed Bliese (2000) procedure. The ANOVA results were non-significant for all variables (i.e., role stressors, LMX, perceived strain, in-role performance, and reward recommendations) and the ICC(1) values did not exceed 0.02 in any case, suggesting that analyses should be conducted at the individual level (LeBreton and Senter, 2008).

### Hypothesis Testing

Table 3 shows the results of multiple regression analyses predicting perceived strain, in-role performance, and reward recommendations, respectively. We first mean-centered predictors before creating the interaction terms (Aguinis and Gottfredson, 2010), then entered all study variables following the same 5-step procedure as in Study 1. As Model 2 shows, controlling for demographics and the other two role stressors, role overload was positively related to perceived strain (*b* = 0.22, *p* < 0.01), thus supporting Hypothesis 1a. Moreover, as Model 6s show, perceived strain was negatively related to in-role performance (*b* = −0.31, *p* < 0.001) and reward recommendations (*b* = −0.17, *p* < 0.05). Hypothesis 1b stated that role overload would be indirectly related to performance (i.e., in-role performance and reward recommendations) through increased strain. This hypothesis was tested using PROCESS (Hayes, 2018). Based on 5,000 bootstrap samples, we found the indirect effect of role overload on in-role performance and reward recommendations through perceived strain to be significantly negative (−0.07, 95% CI = [−0.13, −0.02]; and −0.04, 95% CI = [−0.11, −0.01]; respectively). Hypothesis 1b is thus supported.

**FIGURE 3:**
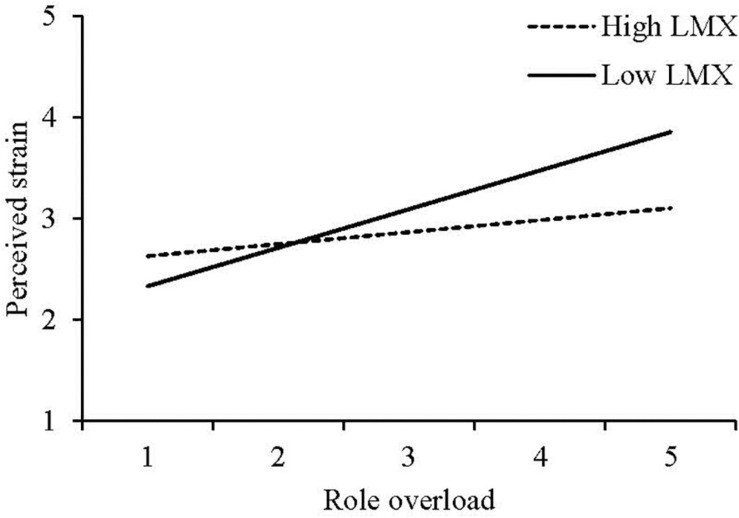
Study 2 Moderating Effect of LMX on the Relationship Between Role Overload and Perceived Strain.

Hypothesis 2a predicted that, controlling for role ambiguity and role conflict, LMX would moderate the positive relationship between role overload and psychological strain, such that this relationship would be less (vs. more) positive at high (vs. low) LMX levels. As Model 5 shows, whereas role ambiguity (b = −0.07, *ns*) and role conflict (b = 0.10, *ns*) did not interact with LMX in predicting perceived strain, role overload did (b = −0.18, *p* < 0.05). To illustrate this interaction, we plotted the regression line for perceived strain on role overload at 1 *SD* below and above the mean of LMX (Figure 3). Role overload was unrelated to perceived strain at high LMX, *t*(190) = 1.06, *ns*, but positively related to it at low LMX, *t*(190) = 3.77, *p* < 0.001, and the slope difference was significant, *t*(190) = −2.33, *p* < 0.05. Hypothesis 2a is thus supported.

Finally, Hypothesis 2b stated that the indirect relationship between role overload and performance through perceived strain would be less (vs. more) negative at high (vs. low) levels of LMX. Using PROCESS on 5,000 bootstrap samples, we found moderated mediation to be significant for in-role performance (0.06, 95% CI = [0.00, 0.15]) and marginally significant for reward recommendations (0.04, 90% CI = [0.00, 0.12]). Moreover, the indirect effect of role overload on in-role performance and reward recommendations through perceived strain was non-significant at high LMX levels (−0.03, 95% CI = [−0.10, 0.04]; and −0.02, 90% CI = [−0.07, 0.01]; respectively) but significantly negative at low LMX levels (−0.12, 95% CI = [−0.22, −0.05]; and −0.08, 90% CI = [−0.17, −0.02]; respectively). These results support Hypothesis 2b.^2^

## Study 2 Discussion

Study 2’s results confirm and extend the findings of Study 1. Using a different measure of psychological strain, we found that role overload was indirectly related to in-role performance and reward recommendations through increased perceived strain. This indicates that psychological strain is a crucial resource-depletion mechanism through which role overload may undermine performance. LMX also buffered the straining effect of role overload, thereby mitigating its detrimental influence on in-role performance and reward recommendations. As expected, the buffering effect of LMX did not apply to role ambiguity and role conflict.

## General Discussion

Using COR theory (Hobfoll, 1989, 2001) as an overarching framework, we conducted two studies that identified psychological strain as a mechanism that explains how role overload leads to reduced performance. Moreover, LMX, as a resource-providing context (Harris et al., 2008), was found to buffer the hindering effect of role overload on strain and performance. These results were obtained while controlling for role ambiguity and conflict and their interactions with LMX. Results from a supplementary study supported our assumption that role overload leads to psychological strain but not vice versa. Combined, findings of our studies help advance the knowledge of how and when role overload is related to work performance.

### Theoretical Implications

Our research contributes to the literature on the trilogy of role stressors in important ways. First, prior research has scarcely examined psychological strain as a mechanism between role overload and reduced performance (for an exception using a work simulation procedure, see Glaser et al., 1999). Rather, research has generally considered job stressors in a broad way, as either challenging or hindering (e.g., Podsakoff et al., 2007), with challenge stressors activating a motivation mechanism and thereby enhancing performance, and hindrance stressors catalyzing a strain mechanism and thus reducing performance (LePine et al., 2005). However, meta-analytic findings indicate that job stressors, whether hindrance or challenge, relate to increased psychological strain (Podsakoff et al., 2007; Mazzola and Disselhorst, 2019). Moving beyond prior research that examined job stressors in a shotgun approach, our research, through the COR lens (Hobfoll, 1989, 2001), zooms in on the trilogy of role stressors—particularly focusing on role overload as a stressor that has presumably challenging and threatening potentials (Eatough et al., 2011). Our primary studies reveal that the threatening component dominates, as shown by role overload triggering psychological strain—ranging from general perceived strain to depression—ultimately undermining performance. This may happen because role overload reflects the perception that role demands exceed personal resources, creating an imbalance featuring more threatening than challenging potentials. This gives rise to a depletion process that drains employees’ resources, which could have been devoted to performance. Our research enriches the understanding of the strain mechanism by considering depression a severe type of strain, which may emerge when role demands greatly exceed personal resources, and further penalizes performance.

Second, our research accentuates the scope of consequences of role overload by examining various aspects of performance (i.e., in-role performance, job dedication, voice behavior, and reward recommendations). In doing so, we demonstrate that if left to their own devices, role overload through triggering a depletion process can inflict detrimental effects on wider performance outcomes than previously thought (Örtqvist and Wincent, 2006; Gilboa et al., 2008; Eatough et al., 2011). Prior research largely examined isolated performance outcomes one at a time like innovation (Montani et al., 2020) and OCBs (Eatough et al., 2011). By assessing multiple performance outcomes, our research confirms that role overload involves many stakes concerning employees’ in-role and extra-role behaviors that may determine their welfare like income and promotion, and as such constitutes a specific role stressor with an array of practical implications.

Third, a worthwhile contribution of this research lies in demonstrating LMX as a potent relational buffer. LMX—by ensuring employees of various resources (e.g., social, psychological, and instrumental) that are readily available—can reduce and even suppress the depleting effects of role overload. Indeed, LMX offers “affective and resource-based support” to employees (Erdogan et al., 2004, p. 311). High LMX may also result in more informal rewards from supervisors (Harris and Kacmar, 2005), hence reduce the depleting effects of role overload. These findings add to prior research that has also addressed LMX’s moderating role, such as in the relationship from perceived politics to depression (Harris and Kacmar, 2005), from general job demands to strain (Hesselgreaves and Scholarios, 2014), from hindrance and challenge stressors to OCBs (Ozer et al., 2014), and from strain to turnover intention (Harris et al., 2008).

While psychological strain likely explains how role overload is associated with reduced performance, LMX describes when such relationship materializes. An essential point is that high-quality LMX was found to reduce, and even suppress, the hindering effects of role overload. Indeed, moderated mediation analyses revealed that the indirect relationship from role overload to performance through strain dropped to non-significance when LMX was high. Given the controversy as to whether role overload comprises both hindrance and challenge components (Gilboa et al., 2008; Eatough et al., 2011), and the meta-analytic finding that all work stressors more strongly relate to increased psychological strain than to enhanced motivation (Mazzola and Disselhorst, 2019), our research suggests that role overload’s hindering potential is not inevitable and can be counteracted by a relational context like LMX.

Finally, the present research indicates differential sensitivity of role stressors to LMX in affecting employee strain and downstream performance. Prior research, by classifying job stressors as either hindrance or challenge factors, examined their global effects on strain and performance (e.g., LePine et al., 2005). This may hide the unique effects associated with each of these stressors. In our research, we accentuate role overload’s effects on psychological strain and subsequently on work performance by controlling for role ambiguity and role conflict. We thus emphasize psychological strain as a unique mechanism linking role overload to work performance. The hindering potential of role overload is further highlighted by its significant interaction with LMX in predicting strain and performance, as opposed to the non-significant parallel interactions between the other two role stressors and LMX. From a COR perspective, LMX acts upon the resource (vs. demand) end of role overload, thereby reducing the resource-demand imbalance and thus preventing role overload from triggering strain and subsequently undermining performance. This may not apply to role ambiguity and role conflict, which represent pure stressors that represent the hindering action of unclear expectations and conflicting demands, respectively (Gilboa et al., 2008, p. 231).

### Practical Implications

Our research confirms that role overload not only harms the individual since it may engender such severe psychological strain as depression, but it can also threaten the organization for it may indirectly undermine work performance—particularly when LMX is low. Therefore, organizations would be well advised to ensure that workload does not exceed individuals’ resources (time, competencies, etc.). Organizations must also be aware that even though some part of an overwhelming workload may come from individuals voluntarily engaging in OCB (Eatough et al., 2011), the net effect of overload is a resource depletion process, which is characterized by lack of energy, lack of pleasure, and reduced quality of life. Nonetheless, our research illustrates that high-quality relationships with one’s supervisor can act as an antidote to the detrimental effects of role overload. Therefore, managers should be aware of their essential role, that is, by establishing a positive relationship with their subordinates, they could reduce and even remove the hindering effect of role overload on work performance, upon which hinges the success of the organization.

Liden and Maslyn (1998) LMX-MDM instrument comprises four dimensions, namely affect, loyalty, contribution, and professional respect, all of which are potential resources that can enable employees to better cope with overwhelming tasks. Affect (i.e., mutual affection between supervisor and employee) strengthens communication; contribution (i.e., the intensity of work-related efforts put into meeting the shared goals) shows that the supervisor is investing resources to develop the subordinate; loyalty (e.g., the public support to the other’s action) provides assurances that the supervisor will support the subordinate; and respect (i.e., a reputation of excellence in one’s work) offers learning opportunities to the subordinate (Dienesch and Liden, 1986; Harris et al., 2008). Managers who take the initiative to turn LMX into a tangible and supportive environment for their subordinates will likely reduce the detrimental effects of role overload.

### Limitations and Directions for Future Research

Our research has limitations. First, we did not address the potential challenge path that might link role overload to performance. Gilboa et al. (2008) and Eatough et al. (2011) suggested that the non-significant association between role overload and work performance, as reported in meta-analytic reviews (Örtqvist and Wincent, 2006; Gilboa et al., 2008; Eatough et al., 2011), possibly hides opposite (indirect) effects on performance. On one hand, role overload might engender negative affective reactions (Fisher, 2014) such as anxiety (Glazer and Beehr, 2005; Perrewé et al., 2005) and lower job satisfaction (Eatough et al., 2011); in this case, role overload is considered a hindrance and as such can harm work performance. On the other hand, employees “may also respond to role overload by increasing their motivation and efforts in order to meet all the demands” (Eatough et al., 2011, p. 626); in this case, role overload is considered a challenge and as such can boost work performance. Thus, the non-significant correlations between role overload and performance in our studies may hide the fact that “competitive mediators” (Zhao et al., 2010) are operating, such as felt job challenge (e.g., Boswell et al., 2004) and motivation (LePine et al., 2005) that can enhance performance while psychological strain exerts the reverse effect. Our study addressed only a straining pathway. A motivating pathway has recently been identified by Montani et al. (2020), who found work engagement to be a resource-based mediator that links workload to innovative behavior.

Second, juxtaposing Montani et al. (2020) study with ours, it seems plausible for future research to test a richer model, in which role overload may bifurcate into two pathways, one straining and the other motivating; in doing so, future research can compare the relative importance of these two pathways (i.e., straining vs. motivating). Third, should motivational mediators be identified, it would be then interesting to determine if LMX moderates the indirect relationship from role overload to performance through these mediators. For example, Ozer et al. (2014) found LMX to amplify the effects of challenge stressors on OCB directed toward the organization. Similarly, if role overload has a challenge component, it would make sense to expect LMX to amplify its indirect effect on performance through increased motivation.

Fourth, other moderators of role overload could be examined. For example, social support from different sources (e.g., co-workers, supervisors, and family and friends) may buffer the effect of role demands on strain (Viswesvaran et al., 1999). Yet, as we studied stressor-strain relations in the work context, work-related sources of support seem more relevant. Moreover, it is unclear whether support from co-workers or family and friends would be as effective as resources provided by supervisors. Indeed, supervisors have the authority to reward employees (which co-workers cannot do) thereby offering employees valuable resources. Notably, Montani et al. (2020) have identified mindfulness as an essential personal resource that can moderate workload’s effects, such that being mindful can not only sustain the motivating pathway but also suppress the straining pathway.

Finally, although the supplementary study used cross-lagged design to examine the longitudinal relationship between role overload and psychological strain, our two-wave panel data did not allow us to holistically test the conditional process model as hypothesized. Future research could use longitudinal designs to track the relationship from role overload to psychological strain and then to work performance over time at different levels of LMX. Such complex longitudinal designs should allow a closer look into how the resource depletion process, once triggered by role overload, unfolds. For example, anxiety can possibly arise as a short-term reaction reflecting high vigilance and activation (e.g., Glazer and Beehr, 2005) and depression may follow as long-term reactions reflecting low vigilance and activation.

## Conclusion

Role overload has a special status in the role-stress literature because the processes and conditions through and under which it relates to work performance remain poorly understood. The present research not only examined a key process, psychological strain, that explains how role overload undermines performance, but also investigated the buffering role of LMX. Combining results from two primary studies, we found psychological strain to be the linchpin linking role overload to underperformance; furthermore, we also found that LMX, a resource-providing context, mitigates the strain pathway through which role overload undermines performance. We hope these results will generate other research endeavors delving into the mechanisms by which role overload relates to work performance.

## Data Availability Statement

The raw data supporting the conclusions of this article will be made available by the authors, without undue reservation, to any qualified researcher.

## Ethics Statement

The studies involving human participants were reviewed and approved by Research Ethics Board at HEC Montreal. The patients/participants provided their written informed consent to participate in this study.

## Author Contributions

W-GT and CV contributed to conception and design of the study. W-GT performed the statistical analysis and wrote the first draft of the manuscript. W-GT and CV contributed to manuscript revision, read, and approved the submitted version. Both authors contributed to the article and approved the submitted version.

## Conflict of Interest

The authors declare that the research was conducted in the absence of any commercial or financial relationships that could be construed as a potential conflict of interest.

## Footnotes

1 Role overload and workload are often used interchangeably in the occupational stress literature (Webster et al., 2011), although some researchers categorize the former as a challenge stressor and the latter a hindrance stressor (Crawford et al., 2010; Mazzola and Disselhorst, 2019).

2 To examine the temporal relation between role overload and psychological strain, we conducted a separate panel study among 99 business alumni (response rate = 81.42%). Average age was 37.95 years (*SD* = 8.35), average organizational tenure was 6.93 years (*SD* = 6.10), average tenure with the supervisor was 2.67 years (*SD* = 2.15), and 50.51% were men. We measured role overload, depression, and perceived strain twice at a 4-month interval (LMX was measured once at Time 1 [T1]), using the same scales as in the primary studies. As depression and perceived strain are thought to reflect the same overall construct of psychological strain, we conducted an exploratory factor analysis to examine the structure of these items using Mplus 8.3. The results suggested that these two variables indicated one construct (explained variance = 55%^*T1*^ and 58%^*T2*^; range of item loadings = [0.64 to 0.83]^*T1*^, and [0.66 to 0.88]^*T2*^). We thus parceled the 12 items (9 for depression and 3 for perceived strain) into 3 indicators of psychological strain (Little et al., 2013). A closer examination showed that role overload and psychological strain were distinguishable [Δχ^2^(1)s = 214.45^*T1*^ and 3299.37^*T2*^ *p*s < 0.001], and displayed good reliability (αs^*T1*^ = 0.93 and 0.94, and αs^*T2*^ = 0.95 and 0.95, respectively) and strict measurement invariance across time (Vandenberg and Lance, 2000). We then built a cross-lagged structural equation model (SEM) to examine the temporal relationship between role overload and strain. We specified four alternative structural models (SM) to examine the cross-lagged relationships (Cole and Maxwell, 2003): SM1 was the stability model with no cross-lagged effects; SM2 was the normal causation model relating T1 role overload to T2 strain; SM3 was the reversed causation model relating T1 strain to T2 role overload; and SM4 was the reciprocal causation model combining SM1, SM2, and SM3. SM2 best described the data, χ^2^(99) = 119.07, CFI = 0.98, TLI = 0.98, RMSEA = 0.05, SRMR = 0.09, and improved over SM1 [Δχ^2^(1) = 4.27, *p* < 0.05], which in turn outperformed SM3 [Δχ^2^(1) = 2.92, *ns*]. Moreover, the fit of SM2 did not differ from the fit of SM4 [Δχ^2^(2) = 2.88, *ns*]. In the retained SM2, T1 role overload was positively related to T2 strain (β = 0.18, *p* < 0.05), controlling for T1 strain (β = 0.56, *p* < 0.001), while T1 strain was unrelated to T2 role overload (β = 0.19, *ns*), controlling for T1 role overload (β = 0.65, *p* < 0.001). These results suggest that role overload temporally preceded psychological strain, which is consistent with the ordering of the variables in Figure 1.
